# Editorial: Advances in the Regulation and Production of Fungal Enzymes by Transcriptomics, Proteomics and Recombinant Strains Design

**DOI:** 10.3389/fbioe.2019.00157

**Published:** 2019-06-28

**Authors:** André Damasio, Gustavo H. Goldman, Roberto N. Silva, Fernando Segato

**Affiliations:** ^1^Department of Biochemistry and Tissue Biology, Institute of Biology, University of Campinas, Campinas, Brazil; ^2^School of Pharmaceutical Sciences of Ribeirão Preto, University of São Paulo, Ribeirão Preto, Brazil; ^3^Ribeirão Preto Medical School, University of São Paulo, Ribeirão Preto, Brazil; ^4^Department of Biotechnology, Engineering School of Lorena, University of São Paulo, Lorena, Brazil

**Keywords:** fungal enzymes, cell factories, genetic engineering, omics, rational design

There are many studies reporting the importance of biological processes related to protein secretion in filamentous fungi including the mechanisms of unfolded protein response (UPR), endoplasmic-reticulum-associated protein degradation (ERAD), lipid biosynthesis, cell wall integrity, vesicles transport, autophagy, kinases among others (Kwon et al., 2014; Malavazi et al., 2014; de Assis et al., 2015; Burggraaf and Ram, 2016; Schalén et al., 2016; Zhang et al., 2016; Yokota et al., 2017).

However, the secretion level of target proteins has been low in general, which lead to the economically unviable production of some enzymes. Due to this fact, it has been desired more robust host strains with higher secretion yield to increase the range of commercial enzymes.

Essentially, the basis of many of the changes underlying strain improvement is either undefined or not in the public domain. After decades of strain improvement programs, details on the genomic evolution of production strains have not been available in scientific literature due to industrial confidentiality.

In general, the production improvement of a specific protein by genetic engineering does not necessarily promote the same effect in the production of other protein. Moreover, the attempts have resulted in the improvement of one target protein secretion instead of total protein secretion. Then, how to achieve the production of industrial strains such as *T. reesei* that produce 120 g/L of proteins? We believe we are far to reach these industrial levels by rational design.

We sincerely thank all researchers who contributed to this Research Topic. Three reviews and five original research articles were published.

The ascomycete *Trichoderma reesei* is one of the main fungal producers of cellulases based on its high production capacity. Fitz et al. reviewed the advances in promoter toolbox for recombinant gene expression in *T. reesei*. The authors discussed established constitutive promoters for gene expression in *T. reesei* such as promoters from *eno1, gpd1*, and *tef1* genes, and also tunable cellulase promoters from *cel6a, cel7a, cel5a*, and *cel12a* genes. Moreover, potential new tunable promoters discovered by transcriptomic studies were explored. The expression of the gene encoding the copper transporter *tcu1* promoter was abolished by the presence of a certain amount of copper in the medium and can be relieved by the addition of a Cu^2+^ chelator. Another promising promoter induced by low amounts of pantothenic acid was validated by fusion with the *T. reesei* ß-glucosidase BGL1.

Emphasizing the importance of transcription factors manipulation in the microbial cell factories field, Alazi and Ram presented an elegant review on the transcriptional regulation of plant biomass-degrading enzymes. A complete survey on rational design of industrial fungal strains with increased or constitutive production of Carbohydrate-Active Enzymes (CAZymes) was shown. Essentially the authors described the constitutive activation of transcription factors and deletion or down-regulation of specific repressors in order to regulate the expression of CAZyme genes. Finally, recent data on chromatin remodeling and CAZymes overexpression as well as the design of synthetic promoters were also reported. Martins-Santana et al. organized an excellent review on systems and synthetic biology tools to redesign metabolic and secondary metabolites pathways in fungal strains.

Two reports focused on yeasts manipulation. Wei et al. described the potential of a high lipid-accumulating strain of *Yarrowia lipolytica* to express a core of cellulase genes. The recombinant strains successfully co-expressed *cbhI, cbhII*, and *egII* from *T. reesei*. In addition, the *Y. lipolytica* recombinant strain showed higher glucose utilization rate that led to ~2-fold more cell mass and 3-fold more lipid production per liter culture compared to parental control strain growing in media with a high C/N ratio. Similarly, Yang et al. expressed cellulases in *Saccharomyces cerevisiae* using the CRISPR-cas9 tool resulting in an improvement in the saccharification of orange-peels.

Midorikawa et al. described the expression of CAZymes by *Aspergillus tamarii* strain BLU37 cultivated on steam-exploded sugarcane bagasse. The results showed a high expression of several genes encoding CAZymes, such as Glycoside Hydrolases (GH), Carbohydrate Esterases (CE), and Auxiliary Activities (AA). Moreover, transcription factors involved in cellulases and hemicellulases regulation were overexpressed such as XlnR and ClrA. Exploring new fungal chassis are fundamental in order to found microbial cell factories with a potential application on industrial enzymes production.

Finally, in this topic two reports used *T. reesei* as a model organism. Borin et al. demonstrated a range of new potential targets to improve the cellulolytic potential of *T. reesei* RUT-C30 strains. Several cellulases, sugar transporters and hypothetical proteins coding genes upregulated in sugarcane bagasse were grouped into different highly connected gene modules. Stappler et al. investigated the influence of different light intensities on cellulase activity and protein secretion by proteomics approach. Several glycoside hydrolases showed light-dependent regulation and the photoreceptor genes *blr1* and *blr2* are important for protein abundance regulation between light and darkness.

## Author Contributions

AD designed and wrote the Editorial with contributions from GG, RS, and FS.

### Conflict of Interest Statement

The authors declare that the research was conducted in the absence of any commercial or financial relationships that could be construed as a potential conflict of interest. The reviewer MQ declared a shared affiliation, though no other collaboration, with several of the authors, GG, RS, and FS to the handling Editor.
